# Association between water, sanitation, and hygiene access and the prevalence of soil-transmitted helminth and schistosome infections in Wolayita, Ethiopia

**DOI:** 10.1186/s13071-022-05465-7

**Published:** 2022-11-04

**Authors:** Anna E. Phillips, Alison K. Ower, Kalkidan Mekete, Ewnetu Firdawek Liyew, Rosie Maddren, Habtamu Belay, Melkie Chernet, Ufaysa Anjulo, Birhan Mengistu, Mihretab Salasibew, Geremew Tasew, Roy Anderson

**Affiliations:** 1 MRC Centre for Global Infectious Disease Analysis, Imperial College London, St Mary’s Campus, London, W2 1PG UK; 2 London Centre for Neglected Tropical Disease Research, London, UK; 3 Ethiopian Public Health Institute, Addis Ababa, Ethiopia; 4 Federal Ministry of Health, Wolayita, Ethiopia; 5 Children’s Investment Fund Foundation, London, WS1 2FT UK

**Keywords:** Soil-transmitted helminths, Schistosomiasis, Water, Sanitation, & Hygiene, WaSH, Interruption of transmission

## Abstract

**Background:**

The Geshiyaro project is a 5-year intervention to assess the impact of community- and school-based water, sanitation, and hygiene (WaSH) interventions on reducing infection with soil-transmitted helminths (STH) and schistosome parasites in combination with deworming in Wolayita zone, Ethiopia.

**Methods:**

A population-based, cross-sectional census and parasitological mapping activity was conducted between 2018 and 2019. Individuals in the census were identified using either a registered study ID card or biometric fingerprint to enable linkage of their household WaSH data with baseline STH and schistosome prevalence for risk analysis.

**Results:**

Prevalence of STH was 15.5% for any STH species, 9.47% for *Ascaris lumbricoides*, 1.78% for *Trichuris trichiura*, and 7.24% for hookworm. Intestinal schistosomiasis (*Schistosoma mansoni*) infection prevalence was 0.85% by Kato Katz, 21.6% by POC-CCA trace positive (Tr +), and 13.3% trace negative (Tr-). Microhaematuria was 2.77%, with 0.13% of people examined with *S. haematobium* eggs detected by urine filtration. At the household level, increased (> 30 min) time taken to collect drinking water, sharing a latrine, and lack of handwashing facilities were all associated with a greater risk of *A. lumbricoides*, hookworm, and *S. mansoni* infection. Not disposing of infant stool at the household and clothes washing/recreational freshwater contact were significantly associated with higher risk of schistosomiasis infection. Aggregating WaSH data at the community level showed odds of *A. lumbricoides,* hookworm, and *T. trichiura* infection were significantly lower as both community sanitation coverage and access to improved drinking water improved.

**Conclusions:**

The principal finding of this study is that lack of access to WaSH, such as improved drinking water and shared toilet and hand-washing facilities, were linked to an increased risk of infection with STH and schistosome parasites. These associations are difficult to establish at an individual household level because of wide variability in access between houses but are detectable when coverage is aggregated at the community level. Maintenance of WaSH facilities as well as increased access within the whole community is important in influencing the community-wide prevalence of infection with STH and schistosome parasites.

**Graphical Abstract:**

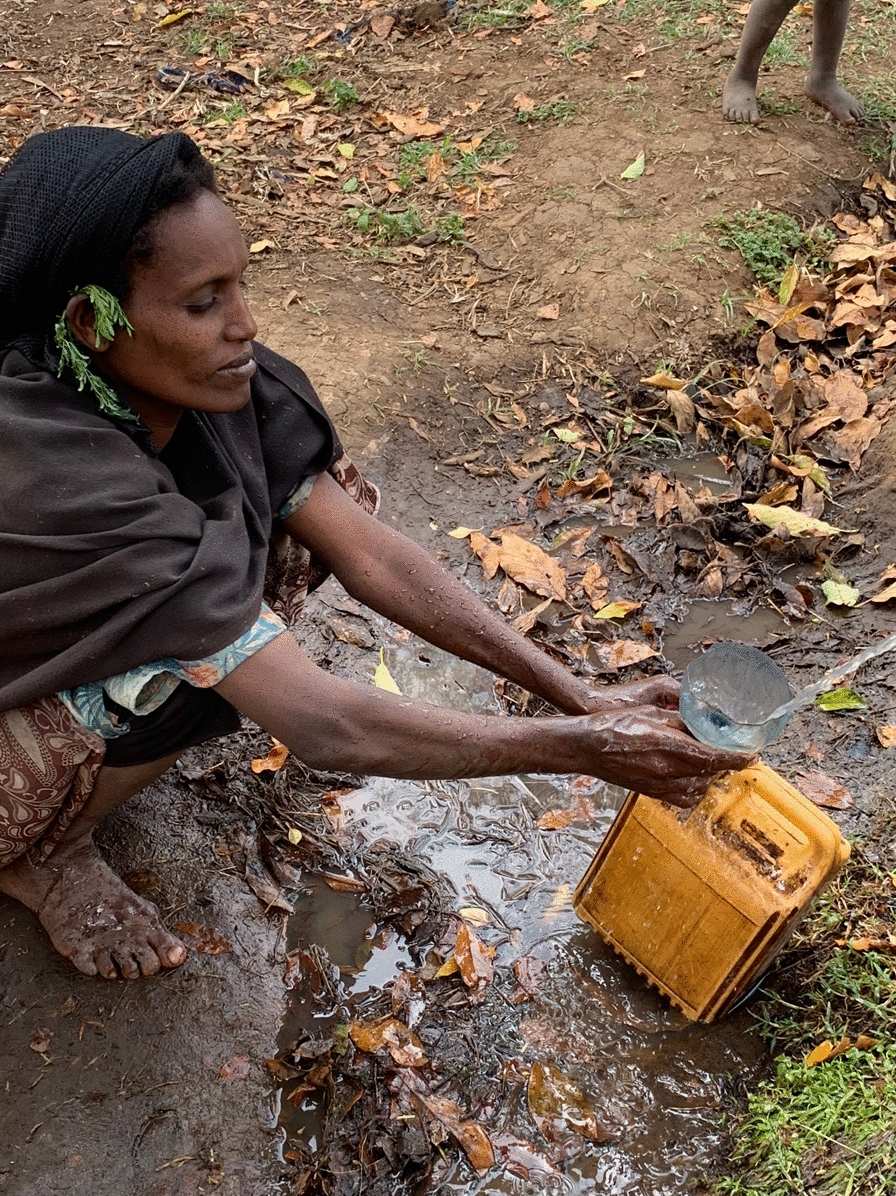

**Supplementary Information:**

The online version contains supplementary material available at 10.1186/s13071-022-05465-7.

## Background

Helminth infections caused by soil-transmitted helminths (STHs) and schistosome parasites (*Schistosoma mansoni* and *Schistosoma haematobium*) are among the most prevalence afflictions of humans who live in areas of poverty in tropical and subtropical areas, with the greatest number occurring in sub-Saharan Africa [1]. According to the World Health Organisation (WHO), > 1.5 billion people, or 24% of the world’s population, are infected with at least one species of STH and an estimated 236.6 million people require preventive treatment against schistosomiasis [2]. Due to the geographic overlap of these parasites and the age-prevalence distribution weighted toward school-aged children (SAC) who exhibit the greatest morbidity, STH and schistosomiasis control often targets this age group [3]. Prolonged infection with these parasites, particularly among children, can lead to school absenteeism, stunted growth, and impaired cognitive development [4–6]. The extent of such long-term sequelae is related to the worm burden harboured by an individual [1]. Most morbidity, however, can be reversed or prevented by periodic preventive chemotherapy (PC) with anthelmintics, usually through school-based platforms, which globally has been the cornerstone control strategy since the early 2000s [7, 8]. Repeated treatment is required since these helminth parasites do not induce strong acquired immunity, such that post-treatment reinfection can rapidly occur. There is debate in the literature on the ability of PC alone to break and sustain the control of helminth infection, although model-based analyses indicate that sustained high levels of coverage can reduce the basic reproductive number of infection (R_0_) to below unity in value [9, 10]. NTDs have multiple routes of transmission, therefore a combination of approaches is ideally required to complement treatment, including improvements in water, sanitation, and hygiene facilities (WaSH).

Helminth infection occurs after either ingesting eggs (*Ascaris lumbricoides* and *Trichuris trichiura*) or larvae penetrating bare skin in contact with the soil (hookworm) or contact with freshwater infested with the infective cercarial stage of the human schistosome parasites. *Ascaris lumbricoides* and *T. trichiura* eggs often survive for several weeks, but can be viable up to several months, whilst hookworm larvae are viable from a few days to several weeks, thus maintaining the environmental transmission beyond the temporal benefits of PC [11]. Likewise for schistosomiasis, re-infection can occur within 18–24 months after treatment if a few infected individuals contaminate the environment containing the intermediate snail host, which in turn can release millions of infective schistosome cercariae into freshwater bodies [12]. As such, without a concurrent change in environmental conditions, such as improvements in sanitation and hygiene behaviours, continued exposure can result in reinfection soon after treatment [13–16].

Some published meta-analyses have found that access to, and use of sanitation facilities, is associated with significantly lower odds of STH [17–19] and schistosome infection [20, 21]. Nonetheless, several randomised controlled trials have reported that despite improvements in infrastructure coverage, household sanitation interventions were insufficient to interrupt environmental contamination and human exposure to STH and schistosomiasis infection [22–34]. Clearly there are challenges in quantifying long-term behavioural change that is not necessarily consistent over time and may be subject to bias in self-reporting (undesirable) behaviour, vary considerably at the individual level within and between households, and present difficulty in measuring access in a community context and understanding how poor maintenance can exacerbate transmission [10, 35–40].

Mathematical modelling suggests the impact of WaSH interventions on STH transmission is species dependent as well as related to the intervention efficacy and the pattern of individual uptake in a defined community [39]. The simulations also indicated that PC could mask the impact of WaSH interventions, but there is a clear added benefit in sustaining the gains made by treatment in the long term via sustained reductions in rates of infection and hence the magnitude of the effective reproductive number, R_t_. These changes reduce the speed of bounce-back of infection to pre-intervention levels.

There are currently no widely accepted standard methods or guidance on how to best design WaSH interventions appropriate for STH and schistosomiasis mitigation, nor on the variables to accurately measure behaviour and facility maintenance. The WHO/UNICEF Joint Monitoring Programme (JMP) for Water Supply, Sanitation and Hygiene provides benchmark service ladders for safe drinking water supply, disposal of human excreta, and handwashing [40]. Since the JMP criteria are not disease-specific indicators, a traffic-light ranking of the JMP service ladders showing the potential transmission risks associated with each tier has been proposed by Campbell et al. [16]. This demonstrates that STH and schistosomiasis need to be considered by their transmission routes, considering terrestrial (STH) or aquatic (schistosomiasis) species, urinary (*S. haematobium*) versus faecal (*S. mansoni* and STH) contamination, and age-infection profiles. Cleanliness and maintenance of sanitation are also not taken into consideration by the JMP guidelines, yet they can exacerbate STH outcomes.

A 5-year Geshiyaro project has been implemented in the Wolayita zone, southwest Ethiopia, investigating the impact of two sets of interventions: community-wide anthelmintic PC and provision of safe water and sanitation alongside behaviour change communication (BCC) on STH and schistosomiasis transmission interruption [41]. The objective of Geshiyaro is to provide evidence to support complementing PC with the provision of WaSH for all. This paper analyses STH and schistosomiasis-specific pre-intervention prevalence and associations with selected WaSH indicators.

## Methods

### Study overview

The Geshiyaro project is being conducted in all 15 districts of Wolayita zone in the Southern Nations and Nationalities People’s region (SNNPR). Details on sampling methods and the project protocol have been published elsewhere [41]. In brief, the objective of the Geshiyaro project is to provide evidence for a scalable, sustainable model of interventions to interrupt the transmission of STH and schistosomiasis through community-wide anthelmintic treatment complemented with WaSH and BCC. This manuscript presents the association of baseline prevalence and intensity of four common STH species: hookworm (*Ancylostoma duodenale* and *Necator americanus*), roundworm (*A. lumbricoides*), and whipworm (*T. trichiura*) and schistosomiasis (*S. mansoni* and *S. haematobium*) with different levels of household and community WaSH access.

### Parasitological data collection

Prevalence and intensity of both STH and schistosomiasis are being evaluated in the Geshiyaro project cross-sectionally at baseline (2018), endline 1 (2023), and endline 2 (2025). In addition, longitudinal sentinel site monitoring of infection in defined cohorts will be conducted in each arm from Year 1 to Year 5. For this analysis, we combined data collected at baseline mapping, which took place between October and December 2018, and the first year of sentinel site surveys in January 2019 and February–March 2020, all pre-treatment.

Baseline mapping took place in 130 communities (approximately 40% of communities in the Wolayita region) randomly selected from 15 districts with a higher proportion of communities in small districts. In each community 100 individuals were surveyed, stratified equally into five age groups: pre-SAC (0–4 years), SAC (5–14 years), adolescents (15–20 years), young adults (21–35 years), and adults 36 + years, equally by sex. Within each selected village, households were chosen using family folders at the village health post. Family folders are created by community health workers and detail the name, date of birth, and sex of every family member in each household in the community. Selected family folders were chosen according to a sampling interval number dependent on the number of households in a community until the desired sample size was reached [42]. In total, 100 family folders/households were selected where as an example in a village of 1000 households every 10th folder was selected. At each household, simple random sampling was used to recruit a single individual from one of five age bands (outlined above) by sex. In communities that had undergone a population census, individuals were identified by their study ID card or biometric fingerprint, enabling linkage of the parasitological sample to the household WaSH data for analysis.

In addition to the cross-sectional mapping, 30 longitudinal sentinel sites were randomly selected from the 130 mapping sites using a stratified framework of low, moderate, and high STH and schistosomiasis prevalence based on mapping results. The sentinel site studies are designed to provide a longitudinal response to any changes to WaSH infrastructure access at each site. In each of the 30 sites, 150 individuals were randomly selected (15 male and females in each of the same five age groups outlined above), resulting in an additional 4500 individuals sampled each year.

### Diagnostics

Each participant provided a stool and urine sample on the day of enrolment. Duplicate thick-smear Kato Katz slides were prepared from each stool sample and read within half an hour of preparation for STH (*A. lumbricoides, T. trichiura*, and hookworm) and *S. mansoni* infection, reported as eggs per gram (epg) [43]. Given the low prevalence of *S. mansoni*, a more sensitive point-of-care circulating cathodic antigen test ((POC-CCA) Rapid Medical Diagnostics, Pretoria, South Africa) was used on the urine sample for further *S. mansoni* investigation. Detection of microhaematuria using urine reagent strips (Hemastix®) was used as a proxy for *S. haematobium*, with urine filtration on haematuria-positive (where trace haemolysed was considered positive) samples for *S. haematobium* egg detection. The urine samples, like the stool, were processed on two separate slides and read by different laboratory technicians. Samples were tracked from collection to diagnostic reading, using barcodes placed on sample pots and microscope slides, and recorded in SurveyCTO software (Dobility, Inc; Cambridge, MA, USA).

### Population census and WaSH survey

**Fig. 1:**
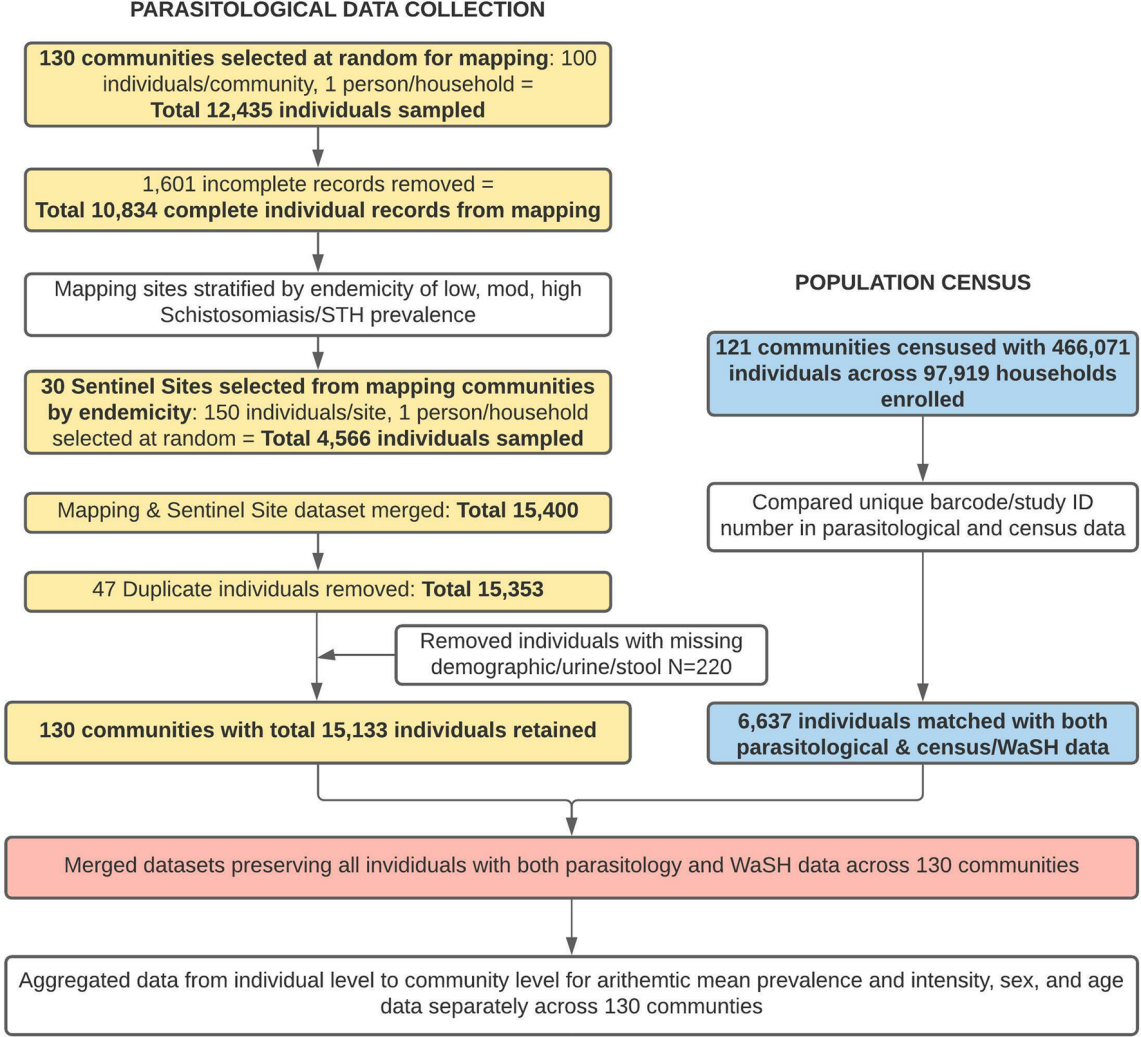
Flow diagram of village and participant numbers

A census of all communities in five of the 15 districts in Wolayita was conducted prior to the start of the intervention. Since approximately 40% of communities in the 15 districts were (randomly) sampled for the mapping, only a subset of individuals had both WaSH and parasitological data. Of the 97,919 individuals who took part in the census, 2236 also provided parasitological samples in the mapping and 4401 individuals in the sentinel sites between October and December 2018 (Fig. 1). The census collected demographic data (age, sex, observed shoe wearing) for all individuals living in the household. Everyone present during the census was registered using a barcoded study ID card, with those aged ≥ 2 years additionally registered using a biometric fingerprint if they provided consent. The head of the household, or other adult decision-maker present, completed a face-to-face interview on the household’s socioeconomic information and practices and access to WaSH infrastructure. Observations on latrine conditions and handwashing facilities were made. All data, including biometric information captured using a Vero fingerprint scanner and the Simprints app, were recorded electronically using SurveyCTO. To maximise enrolment coverage, enumerators returned up to three times to mop-up biometric registrations from those previously missed or declined. The unique biometric and/or study ID card identifiers enabled the linking of individual census information with parasitological data and, in future, treatment compliance.

### Data analysis

All analyses were performed in Stata 16 (College Station, TX, USA). The outcome of interest in this analysis was prevalence (proportion infected) and intensity of infection for STH and schistosomiasis species. One participant was removed from the analysis because of a high *A. lumbricoides* intensity, 9,694,296 epg, which appears to be a counting or data entry error. All *S. haematobium* associations are based on diagnosis by Haemastix (as a proxy of infection) and not urine filtration as only haematuria-positive samples were filtered. Infection intensity was calculated using the arithmetic mean epg value, using both egg positive and negative values (the full probability distribution of intensity measure in the sampled population), across the means of duplicate Kato-Katz smears [2]. The exposure variable was household and community WaSH (sanitation, water, and hygiene) coverage. Household access to water was dichotomized as improved, or not, according to WHO/UNICEF JMP classification [40]. Drinking water was further classified into three categories: none, limited, or basic service (Table 1). Basic water access was defined as an improved drinking water source, available in a round trip (walking each way, plus any additional wait time) of < 30 min at time of the survey. Type of latrine was recorded, which was further categorized as none, limited, or basic service per JMP guidelines at the time of the interview. Basic hygiene was defined as access to handwashing facilities, with both water and soap observed at the time of the survey. The questionnaire also incorporated a question on exposure to surface water for bathing, laundry, and playing to capture contact with freshwater bodies, relevant for schistosomiasis transmission.

**Table 1:** JMP WaSH service ladders categories used for household WaSH anaylsis [40]

Service level*	Drinking water	Sanitation	Hygiene
Improved	Improved water sources, includes piped water, boreholes or tubewells, protected dug wells, protected springs, rainwater, or packaged water	Facilities that hygienically dispose of excreta: flush/pour flush toilets connected to sewer systems, septic tanks, pit latrines with slabs (including ventilated pit latrines), and composting toilets	
Basic	Improved source and collection time < 30 min round trip from household	Use of improved facilities and not shared with other households	Availability of handwashing facility with soap (bar, liquid, or powder detergent) and water available at household at the time of the survey
Limited	Drinking water from an improved source and collection time > 30 min round trip from household	Use of improved facilities and shared with other	Availability of a handwashing facility without soap and/or water at household at the time of the survey
Unimproved	Drinking water from unprotected dug well or unprotected spring	Use of pit latrines without a slab or platform, hanging latrines or bucket	
No service	Surface water: Drinking water directly from a river, dam, lake, pond, stream, canal	Open defecation: Disposal of human faeces in open places	No handwashing facility or no water at household

^*^Safely managed for drinking water or sanitation is not included as data on contamination of drinking water were not available or sewage removal for sanitation

Prevalence estimates include all samples with 95% confidence intervals using Taylor linearization method to account for clustering within communities to account for study design and unequal selection probability. Univariate analysis of association between explanatory variables (age, sex, WaSH access) and binary outcome (infected or not) was carried out accounting for survey design using SVY method in STATA. F-statistics were calculated using adjusted Wald test for categorical variables and ANOVA for continuous variables. The level of statistical significance was set at *P* < 0.05. To assess whether any WaSH factors were associated with STH/schistosomiasis prevalence or influenced by multiple factors, a mixed effects logistic regression model was performed. To select variables for multivariable analysis, any variable with *P* > 0.05 in the univariate analysis was pre-specified in a sequential (block-wise) variable selection method and included in the backward logistic regression model. Sex and age were retained as fixed terms in the final model regardless of statistical significance because of their known importance. Multilevel analysis to assess the influence of community-level effects on an individual outcome was used in addition to between- and within-group variability. The effect of WaSH on intensity of infection was analysed using a multilinear regression model, assuming a negative binomial distribution of intensity measures with a log link of egg counts [10]. Confounding covariates included in the multivariate analysis were sex, age, and (observed) shoe wearing.

## Results

### Parasitological data

Of the 15,400 samples obtained at both baseline mapping and sentinel site surveys, parasitological data were obtained for 15,133 individuals across 130 communities in 15 districts.

**Fig. 2:**
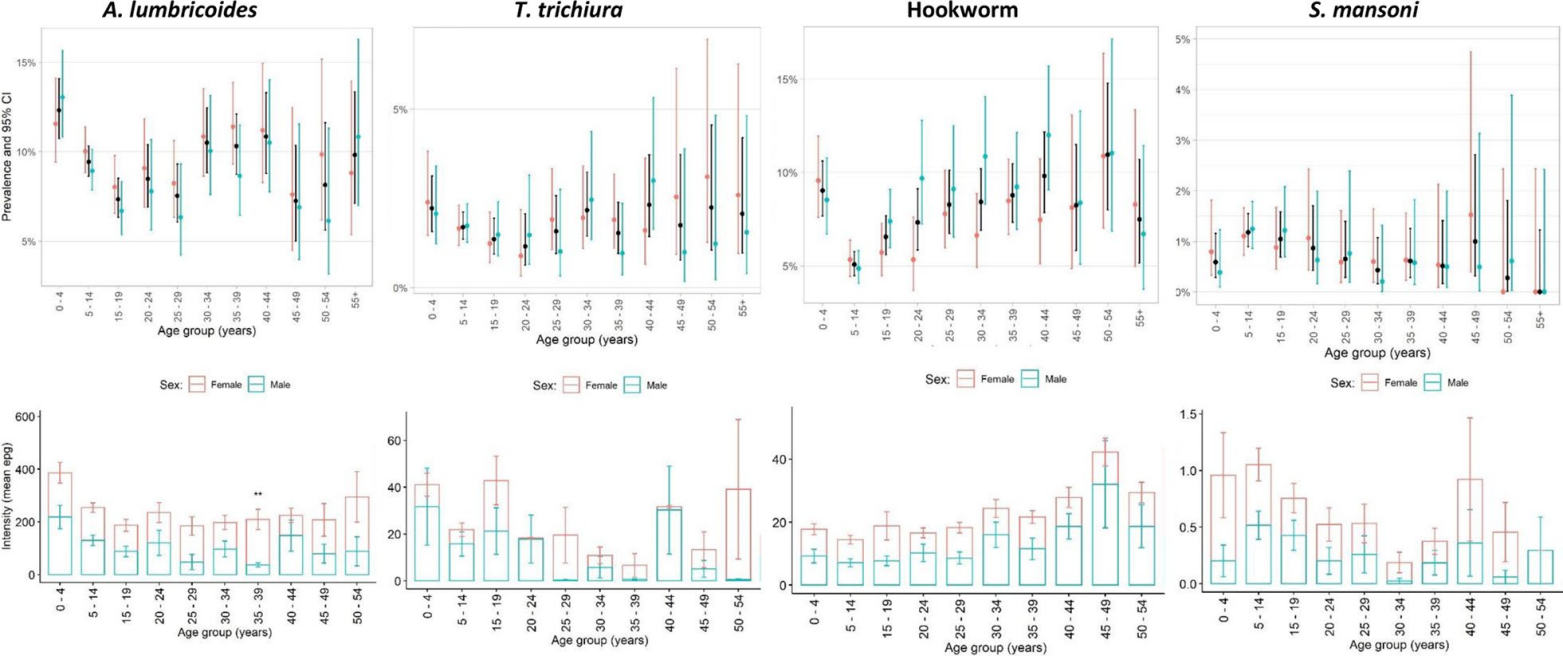
Prevalence of soil-transmitted helminths and schistosomiasis by age group

Overall, prevalence with at least one STH was 15.5%, with considerable heterogeneity between communities (0–61.0%) as is commonly observed in epidemiological studies of helminth infections. The most prevalent species was *A. lumbricoides* (9.47%), followed by hookworm (7.24%) and *T. trichiura* (1.78%) (Table 2). The percentage of moderate infections was low across all species with 7.54% of *A. lumbricoides*, 18.96% for *T. trichiura*, and 0.55% for hookworm. There was only one heavy-intensity STH infection (*T. trichiura*). *Ascaris lumbricoides* and to a lesser extent *T. trichiura* infection was highest in pre-SAC (Fig. 2). Prevalence of hookworm typically increased with age, with the exception of an additional peak in pre-SAC (*P* > 0.01) (see Table 2; Fig. 2). Mean intensity was very low, reflecting the impact of previous predominantly school-based PC. As such, the sampled communities displayed aggregated distribution of worm burden whereby most people were uninfected or harboured a low intensity infection, whilst a minority harboured moderate intensity infections that can enable continued transmission within communities.

**Table 2:** Bivariate analysis of STH infection by respondent characteristics

Characteristics	Total N (%)	*A. lumbricoides* (n = 1433) % (95% CI)	P*	*T. trichiura* (n = 269) % (95% CI)	P*	Hookworm (n = 1095) % (95% CI)	P*	Any STH (n = 2348) % (95% CI)	P*
Male	7249 (48.9)	8.9 (8.2, 9.5)	0.05	1.7 (1.4, 2.0)	0.76	7.6 (7.0, 8.2)	0.07	15.5 (14.7, 16.3)	0.72
Female	7719 (51.1)	9.8 (9.7, 9.9)		1.8 (1.5, 2.1)		6.8 (6.3, 7.4)		15.3 (14.5, 16.1)	
Age group (years)									
0–4	1527 (10.1)	12.8 (10.8, 14.1)	< 0.01	2.2 (1.6, 3.1)	0.73	9.1 (7.7, 10.6)	< 0.01	20.4 (18.4, 22.5)	0.30
5–14	4660 (30.8)	9.4 (8.6, 10.3)		1.7 (1.4, 2.1)		5.1 (4.5, 5.8)		13.4 (12.4, 14.4)	
15–20	2760 (18.2)	7.9 (6.9, 8.9)		1.4 (1.0, 1.9)		6.7 (5.8, 7.7)		13.3 (12.1, 15.0)	
21–35	3332 (22.0)	9.1 (8.2, 10.1)		1.7 (1.3, 2.2)		8.1 (7.2, 9.0)		16.0 (14.8, 17.3)	
> 36	2686 (17.8)	9.4 (8.3, 10.5)		1.9 (1.4, 2.4)		9.3 (8.3, 10.5)		17.2 (15.9, 18.7)	
Total		9.5 (9.0, 10.0)		1.8 (1.6, 2.0)		7.2 (6.8, 7.7)		15.5 (14.9, 16.1)	

Estimates weighted to adjust for non-response/non-availability of data on STH infections; 95% CI calculated using Taylor linearization method to account for clustering within communities

*CI* confidence interval, *STH* soil-transmitted helminths

^*^p values were calculated using Cochran-Mantel-Haenszel statistics (based on table scores)

^**^Arithmetic mean intensity calculated for those positive only

Overall, the prevalence of infection for intestinal schistosomiasis (*S. mansoni*) was low at 0.85% (mean epg 0.76) by Kato-Katz, 13.1% by POC-CCA when trace results were considered negative (trace-negative, Tr-) and 21.6% when considered positive (trace-positive, Tr +) (Table 3). Only 11 (8.47%) moderate or heavy intensity infections were observed. Microhaematuria was 2.77%, and in 0.13% of samples (20 individuals) eggs were confirmed by urine filtration. Given the low prevalence by urine filtration, only Haemastix results are presented in the tables. Both *S. mansoni* (by POC-CCA) and *S. haematobium* (by Haemastix) prevalences were significantly higher in males compared to females and in SAC when compared to adults (see Table 3; Fig. 2). The age-infection peak for *S. mansoni* was in the 5–9 years age group, albeit with a low peak intensity (0.64 mean epg).

**Table 3:** Bivariate analysis of schistosomiasis by respondent characteristics

Characteristics	Total N (%)	*S. mansoni* by Kato-Katz (*n* = 129) % (95% CI)	P*	*S. mansoni* POC-CCA tr + (*n* = 3,251) % (95% CI)	P*	*S. mansoni* POC-CCA tr- (*n* = 1,867) % (95% CI)	P*	Haemastix positive ‡(*n* = 411) % (95% CI)	P*
Male	7249 (48.9)	0.9 (0.7, 1.1)	0.86	22.6 (21.0, 24.4)	< 0.01	14.5 (13.0, 16.1)	< 0.01	2.4 (2.1, 2.8)	0.02
Female	7719 (51.1)	0.8 (0.7, 1.1)		18.1 (16.5, 19.8)		11.5 (10.2, 12.9)		3.1 (2.7, 3.5)	
Age group (years)									
0–4	1527 (10.1)	0.6 (0.3, 1.1)	0.04	22.6 (19.7, 25.7)	< 0.01	13.1 (10.8, 15.7)	< 0.01	2.4 (1.7, 3.3)	0.02
5–14	4660 (30.8)	1.9 (0.9, 1.5)		25.2 (22.7, 27.9)		17.5 (15.3, 19.9)		2.3 (1.9, 2.8)	
15–20	2760 (18.2)	1.0 (0.7, 1.4)		23.2 (20.2, 26.5)		16.4 (13.8, 19.4)		3.0 (2.4, 3.7)	
21–35	3332 (22.0)	0.6 (0.4, 0.9)		17.3 (15.0, 19.9)		10.0 (8.24, 12.2)		3.0 (2.5, 3.7)	
> 36	2686 (17.8)	0.6 (0.4, 1.0)		12.8 (10.7, 15.2)		7.47 (5.9, 9.5)		3.2 (2.6, 3.9)	
Total	14,968	0.9 (0.5, 1.6)		21.6 (20.2–21.7)		13.3 (12.6, 13.6)		2.8 (2.3, 3.4)	

Estimates weighted to adjust for non-response/non-availability of data on infections, 95% CI calculated using Taylor linearization method to account for clustering within communities

*CI* confidence interval, *Tr + * trace positive, *Tr −* trace negative

^‡^Based on Haemastix test for blood in urine as proxy for infection. *Schistosoma haematobium*-related findings are based on Haemastix-positive results and not UF

^*^Arithmetic mean intensity calculated for those positive only

### Population census and WaSH survey

Overall, 121 communities conducted a population census in a subset of five out of the 15 districts that were mapped for STH and schistosomiasis. Of these 121 communities, a subset of 44 communities also participated in both the census and parasitological survey; therefore 6,637 individuals were able to have their household WaSH data linked to their prevalence data (Fig. 2).

Table 4 describes household WaSH access of the survey respondents by STH species. Most households (78.7%) accessed water from a public standpipe (54.7%). Access to improved drinking water was not statistically associated with STH infection. Of those households that did have access to improved drinking water, only half (50.7%) were able to collect water in < 30 min round trip. Time taken to collect water did have a significant association with *A. lumbricoides* and hookworm, where infection prevalence was significantly higher in households that had to walk > 30 min (14.7% vs. 12.6% and 12.2% vs. 8.50%, respectively). The JMP service ladder for drinking water (Table 1) considers both source and collection time. There was no association between JMP drinking water categories and STH. Reported treatment of household water with chlorine was significantly associated with reduced *A. lumbricoides, T. trichiura*, and hookworm infection.

**Table 4:** Bivariate analysis of STH infection by household WaSH access

Characteristics	Total N (%)	*A. lumbricoides* (*n* = 1433) % (95% CI)	p*	*T. trichiura* (*n* = 269) % (95% CI)	p*	Hookworm (*n* = 1095) % (95% CI)	p*	Any STH (*n* = 2348) % (95% CI)	P*
Household drinking water source*** (*n* = 6637)									
Has improved drinking water Yes No	5201 (78.7) 1408 (21.3)	13.5 (12.6, 14.4) 14.2 (13.3, 15.1)	0.47	2.7 (2.3, 3.2) 2.5 (2.1, 2.9)	0.64	10.6 (9.8, 11.4) 9.3 (8.6, 10.0)	0.16	22.7 (21.6, 23.9) 22.4 (21.3, 23.6)	0.83
< 30 min to water Yes No	3323 (50.7) 3314 (49.9)	12.6 (11.5, 13.8) 14.7 (13.4, 16.1)	0.01	2.4 (1.9, 3.1) 2.9 (2.4, 3.5)	0.20	8.5 (7.7, 9.3) 12.2 (11.1, 13.3)	< 0.01	22.0 (20.7, 23.4) 23.4 (22.0, 24.9)	0.17
Basic Limited Unimproved Surface water	2716 (41.1) 2485 (37.6) 832 (12.6) 576 (8.72)	12.6 (11.4, 13.9) 14.5 (13.1, 15.9) 13.8 (11.6, 16.3) 14.8 (12.1, 17.9)	0.20	2.9 (2.3, 3.6) 2.5 (2.0, 3.2) 2.2 (2.0, 3.4) 3.0 (1.8, 4.0)	0.19	12.4 (11.2, 13.7) 8.6 (7.5, 9.7) 9.5 (7.6, 11.7) 9.0 (6.9, 11.7)	0.06	23.6 (22.0, 25.2) 21.7 (20.2, 23.4) 21.8 (19.1, 24.7) 23.4 (20.2, 27.1)	0.36
Treats water with chlorine Yes No	147 (7.1) 1930 (92.9)	8.2 (6.8, 9.6) 15.7 (13.0, 19.0)	< 0.01	0.7 (0.6, 0.8) 4.3 (3.6, 5.0)	< 0.01	4.7 (3.9, 5.5) 9.2 (7.3, 11.1)	< 0.01	12.9 (10.7, 15.1) 24.5 (20.3, 28.7)	< 0.01

Characteristics	Total N (%)	*A. lumbricoides* (n = 1433) % (95% CI)	P*	*T. trichiura* (n = 269) % (95% CI)	P*	Hookworm (n = 1095) % (95% CI)	P*	Any STH (n = 2348) % (95% CI)	P*
Observed household sanitation facilities*** (*n* = 6637)									
Has improved latrine Yes No	1058 (15.9) 5579 (84.1)	12.9 (11.1, 15.1) 13.8 (11.9, 15.1)	0.46	1.9 (1.3, 3.0) 2.8 (1.8, 4.3)	0.14	9.7 (8.1, 11.7) 10.4 (8.7, 12.5)	0.48	21.4 (19.0, 23.9) 23.0 (20.4, 25.7)	0.26
Has access to shared latrine Yes No	1227 (18.5) 4896 (73.8)	16.2 (14.3, 17.5) 13.1 (10.8, 14.8)	0.02	3.0 (2.4, 4.1) 2.6 (1.6, 4.1)	0.57	11.1 (9.3, 13.1) 8.6 (7.0, 10.6)	< 0.01	24.3 (21.9, 26.8) 22.7 (20.1, 25.4)	0.06
Basic Limited Unimproved Open defecation	770 (11.6) 288 (4.3) 5065 (76.3) 514 (7.8)	13.0 (10.8, 15.6) 12.9 (9.4, 17.2) 13.9 (13.0, 14.9) 12.8 (10.2, 16.0)	0.80	2.0 (1.2, 3.2) 2.1 (0.9, 4.6) 2.9 (2.4, 3.4) 2.1 (1.2, 3.8)	0.09	10.4 (8.4, 12.8) 8.0 (5.4, 11.7) 10.8 (9.9, 11.7) 7.2 (5.3, 9.8)	0.04	21.6 (18.8, 24.6) 20.8 (16.5, 25.9) 23.4 (22.2, 24.5) 19.1 (15.9, 22.7)	0.10
Child stool disposed of**** Yes No	1892 (86.8) 229 (10.5)	14.7 (9.8, 20.5) 14.9 (10.0, 20.7)	0.76	3.3 (1.7, 3.0) 2.2 (1.2, 2.0)	0.43	9.1 (7.5, 11.1) 7.9 (6.0, 10.4)	0.80	22.2 (19.4, 25.2) 19.7 (16.5, 23.3)	0.48

Characteristics	Total N (%)	*A. lumbricoides* (*n* = 1433) % (95% CI)	p*	*T. trichiura* (n = 269) % (95% CI)	p*	Hookworm (n = 1095) % (95% CI)	p*	Any STH (n = 2348) % (95% CI)	P*
Observed household handwashing facilities*** (*n* = 6637)									
Handwashing facility < 3 m from latrine Yes No	1,059 (23.5) 3,440 (76.5)	15.2 (8.5, 23.0) 13.2 (7.4, 20.0)	0.10	2.3 (1.7, 2.8) 2.9 (2.1, 3.5)	0.40	7.8 (6.0, 10.3) 12.3 (10.6, 16.2)	< 0.01	21.5 (16.1, 27.9) 24.2 (18.8, 30.6)	0.08
Water & soap at handwashing facility Yes No	257 (24.2) 484 (75.7)	10.5 (8.6, 13.5) 14.5 (13.3, 15.3)	0.21	3.1 (2.2, 4.6) 4.7 (3.8, 6.1)	0.91	6.2 (4.3, 8.6) 8.9 (7.0, 11.3)	0.19	17.1 (15.2, 19.3) 21.9 (19.5, 24.7)	0.01
Basic Limited No facility	574 (12.8) 935 (20.8) 2990 (66.4)	9.6 (7.4, 12.3) 13.7 (12.5, 14.5) 16.3 (14.7, 18.7)	< 0.01	2.0 (1.2, 3.7) 2.5 (1.6, 3.7) 2.9 (2.4, 3.6)	0.20	7.1 (5.3, 9.6) 7.3 (5.8, 9.6) 13.2 (12.0, 14.5)	< 0.01	16.7 (6.8, 11.8) 22.0 (19.5, 24.8) 25.3 (24.8, 26.9)	< 0.01
Shoe wearing at interview Yes No	2512 (12.5) 4143 (27.4)	12.9 (11.7, 14.3) 14.1 (12.9, 14.9)	0.18	2.9 (2.4, 3.7) 2.5 (1.6, 3.7)	0.26	8.3 (7.3, 9.4) 11.5 (10.1, 13.0)	< 0.01	19.8 (18.3, 21.4) 24.4 (21.9, 27.2)	< 0.01

*P values were calculated using Cochran-Mantel-Haenszel statistics (based on table scores)

***Improved sources based on definitions established by the UNICEF and WHO Joint Monitoring Program (see Table 1)

Few households (15.9%) had an improved latrine, which made it difficult to analyse association with STH infection. Most households (76.1%) had pit latrines without a slab; some reported no facility at all (7.7%). Households were asked if they had access to a shared latrine (i.e. not a private latrine), which was significantly associated with greater *A. lumbricoides* and hookworm infection (16.2% vs. 13.1% and 11.1% vs. 8.6%, respectively). Sanitation was categorised into the JMP service ladder (Table 1), which considers latrine type and whether it is shared with other households. There was a significant association between unimproved sanitation and increased hookworm infection (*P* = 0.04). Most households with infants reported disposing of faeces in the latrine (68.5%). There was no statistical association between infant stool disposal practices and STH.

Few households had observed handwashing facilities within 3 m of the household latrine (23.5%), of which even fewer had soap (3.87% of all households). Only hookworm was significantly greater among households with no handwashing facilities, with 12.3% infection in households without a place to wash hands vs. 7.8% in households with facilities. Access to soap at the handwashing facility was low (24.2%) and associated with lower infection with any STH (i.e. non-species specific). Handwashing was categorized into the JMP service ladder (Table 1), which considers availability of water and soap to wash hands at the time of the survey. The lack of any handwashing facility was significantly associated with increased *A. lumbricoides*, hookworm, and any STH infection (*P* < 0.01). As expected, hookworm infection was significantly associated with shoe wearing, where individuals observed to be wearing shoes at the time of interview were less likely to be infected (*P* < 0.01).

### Association between schistosomiasis and household WaSH

Table 5 describes household WaSH access of the survey respondents by schistosomiasis. Households without access to improved drinking water sources were more likely to have individuals infected with *S. mansoni* considering POC-CCA Tr- (16.2% vs. 13.2%). As with STH, there was higher risk of *S. mansoni* infection when it took the household > 30 min round trip to collect water. As expected, there was lower risk of any schistosomiasis where households had access to basic drinking water (improved source < 30 min round trip). Treatment with chlorine had a significant association with reduced *S. mansoni* (by POC-CCA Tr +) infection (*P* = 0.05).

**Table 5:** Bivariate analysis of schistosomiasis infection by household WaSH access

Characteristics	Total N (%)	*S. mansoni* by Kato-Katz (*n* = 129) % (95% CI)	*p*	*S. mansoni* POC-CCA tr + (*n* = 3251)% (95% CI)	*p*	*S. mansoni* POC-CCA tr− (*n* = 1867) % (95% CI)	*p*	Haemastix positive ‡(*n* = 411) % (95% CI)	*P*
Household drinking water source** (*n* = 6637)									
Has improved drinking water *Yes* *No*	5,201 (78.7) 1,408 (21.3)	1.7 (1.3, 2.0) 1.2 (0.8, 1.5)	0.19	20.9 (19.5, 22.2) 19.8 (18.4, 21.1)	0.14	13.2 (12.5, 14.4) 16.2 (15.3, 17.7)	< 0.01	3.1 (2.7, 3.6) 2.5 (1.8, 3.4)	0.10
< 30 min to water Yes No	3,323 (50.7) 3,314 (49.9)	1.0 (0.7, 1.4) 2.1 (1.5, 2.6)	< 0.01	16.5 (15.0, 18.2) 23.9 (21.7, 26.3)	< 0.01	9.7 (8.5,11.1) 16.1 (15.2, 17.6)	< 0.01	3.0 (2.5, 3.7) 2.7 (2.2, 3.4)	0.52
Basic Limited Unimproved Surface water	2,716 (41.1) 2,485 (37.6) 832 (12.6) 576 (8.72)	0.9 (0.6, 1.3) 2.5 (0.6, 3.3) 1.3 (0.7, 3.2) 1.0 (0.5, 2.3)	< 0.01	16.9 (15.2, 18.8) 24.8 (22.8, 27.0) 14.8 (11.7, 18.6) 19.2 (15.5, 23.6)	0.06	9.6 (8.3, 11.1) 16.8 (15.1, 18.7) 9.7 (7.2, 13.0) 12.5 (9.5, 16.4)	0.01	3.10 (2.5, 3.8) 3.10 (2.5, 3.9) 1.38 (0.8, 2.5) 2.51 (1.5, 4.2)	0.05
Treats water with chlorine Yes No	147 (7.1) 1,930 (92.9)	3.4 (1.9, 4.5) 2.4 (1.5, 4.1)	0.08	19.1 (15.1, 23.5) 16.1 (14.6, 18.0)	0.05	10.9 (8.4,14.2) 9.6 (7.1, 12.9)	0.19	0.7 (0.6, 0.8) 0.6 (0.5, 0.7)	0.63

Characteristics	Total N (%)	*S. mansoni* by Kato-Katz (*n* = 129) % (95% CI)	*p*	*S. mansoni* POC-CCA tr + (*n* = 3251)% (95% CI)	*p*	*S. mansoni* POC-CCA tr− (*n* = 1867) % (95% CI)	*P*	Haemastix positive ‡(*n* = 411) % (95% CI)	*P*
Observed household sanitation facilities** (*n* = 6637)									
Has improved latrine Yes No	1058 (15.9) 5579 (84.1)	1.5 (0.9, 2.5) 1.6 (0.8, 2.4)	0.88	17.4 (14.4, 20.7) 13.0 (11.0, 15.2)	0.15	12.9 (10.4, 15.8) 13.1 (10.2, 15.6)	0.93	2.4 (1.6, 3.5) 1.2 (0.5, 1.7)	0.06
Has access to shared latrine Yes No	1227 (18.5) 4896 (73.8)	1.5 (0.9, 2.5) 1.6 (0.8, 2.4)	0.88	19.4 (16.4, 22.7) 13.0 (11.0, 15.2)	< 0.01	12.9 (10.4, 15.8) 13.1 (10.2, 15.6)	0.93	4.1 (3.7, 4.5) 2.7 (2.4, 3.0)	< 0.01
Basic Limited Unimproved Open defecation	770 (11.6) 288 (4.34) 5065 (76.3) 514 (7.74)	1.0 (0.5, 2.1) 2.8 (1.4, 5.5) 1.7 (1.3, 2.1) 0.8 (0.3, 2.1)	0.09	15.6 (12.3, 19.6) 26.0 (20.6, 32.3) 20.6 (19.2, 22.0) 18.8 (14.2, 24.4)	0.13	9.1 (6.6, 12.4) 19.5 (14.8, 25.4) 13.0 (11.9, 14.2) 13.1 (9.30, 18.1)	0.44	2.6 (1.7, 4.0) 1.8 (0.8, 4.3) 3.1 (2.7, 3.7) 1.2 (0.6, 2.7)	0.07
Child stool disposed of Yes No	1892 (86.8) 229 (10.5	1.3 (1.0, 1.8) 2.6 (1.3, 5.4)	0.47	16.2 (12.9, 20.2) 22.4 (21.0, 23.8)	0.03	9.6 (7.1, 12.9) 13.8 (12.7, 15.0)	0.12	2.0 (1.7, 2.4) 3.2 (2.8, 3.6)	0.03

Characteristics	Total N (%)	*S. mansoni* by Kato Katz (*n* = 129) % (95% CI)	*P*	*S. mansoni* POC-CCA tr + (*n* = 3251) % (95% CI)	*P*	*S. mansoni* POC-CCA tr− (*n* = 1867) % (95% CI)	*P*	Haemastix positive ‡(*n* = 411) % (95% CI)	*P*
Observed household handwashing facilities** (*n* = 6637)									
Handwashing < 3 m from latrine Yes No	1,059 (23.5) 3,440 (76.5)	2.5 (1.2, 5.3) 1.6 (1.3, 2.l)	0.06	24.8 (23.2, 26.5 20.2 (18.6, 21.9)	0.01	17.3 (14.3, 19.0) 13.1 (11.7, 14.5)	< 0.01	3.9 (3.4, 6.1) 2.6 (2.1, 3.3)	0.03
Basic Limited No facility	574 (12.8) 935 (20.8) 2,990 (66.4)	1.9 (1.1, 3.4) 1.9 (1.2, 3.0) 1.7 (1.3, 2.2)	0.88	18.2 (14.1, 23.1) 25.5 (22.4, 28.8) 20.5 (18.9, 22.2)	0.01	12.2 (8.91, 16.6) 18.1 (15.5, 21.1) 13.2 (11.8, 14.6)	< 0.01	0.9 (0.4, 2.2) 4.2 (3.1, 5.8) 2.9 (2.3, 3.6)	0.05
Exposure to surface water (*n* = 6637)									
Uses surface water for recreational use ∞ Yes No	795 (38.3) 1282 (61.7)	3.1 (2.5, 3.6) 2.0 (1.7, 2.4)	0.01	16.3 (13.1, 19.1) 14.2 (10.1, 18.1)	0.06	9.9 (8.4, 11.6) 9.3 (7.8, 11.0)	0.69	0.7 (0.6, 0.8) 0.3 (0.2, 0.4)	0.27
Uses surface water for clothes washing Yes No	1188 (27.1) 2,077 (72.9)	2.7 (2.4, 3.1) 1.3 (0.9, 1.8)	0.01	13.7 (11.8, 15.9) 10.5 (8.3, 12.7)	0.05	8.6 (6.1, 11.0) 5.8 (3.8, 7.7)	0.05	0.5 (0.4, 0.6) 0.3 (0, 1.6)	0.27

**Defined by the UNICEF and WHO Joint Monitoring Program (see Table 1)

***N = 411 Haematuria-positive samples

^**‡**^*N* = 20 Urine filtration positive. These are the intensity counts for the UF positive samples only

∞Recreational water contact is defined as swimming and/or bathing

There was no association with access to an improved latrine; however, sharing a latrine did have a significant association with increased *S. mansoni* (by POC-CCA Tr +) and *S. haematobium* (by Haemastix) (19.4% vs. 13% and 4.1% vs. 2.7%, respectively; *P* < 0.01). If a household did not report disposing of child stool, there was a significant association with *S. mansoni* (by POC-CCA Tr +) and *S. haematobium* (by Haemastix).

Schistosome infections were significantly greater in households with limited handwashing facilities (no soap and/or water available at the time of the survey). There was no association between use of fresh water for recreational use/clothes washing with *S. mansoni*.

**Fig. 3:**
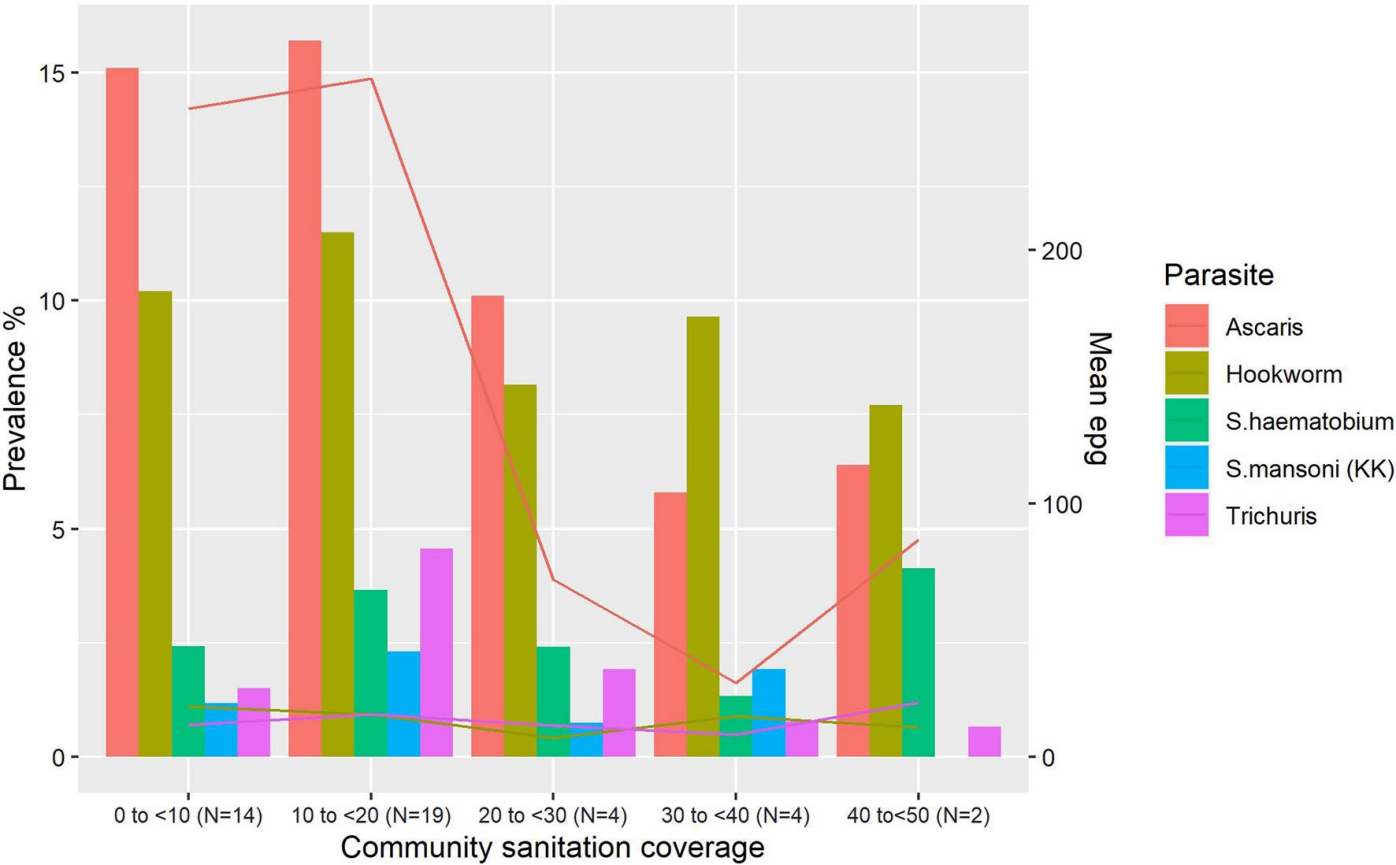
Prevalence of STH and schistosomiasis infection by community sanitation coverage

Figure 3 (and Additional file 1: Table S1) summarises the association between sanitation aggregated at the community level and parasitological results. No communities had a community-level average of improved sanitation coverage > 50%. In general, individuals living in communities with low sanitation usage (particularly < 20%) had a higher prevalence and intensity of STH infection. There was no statistically significant effect of community sanitation coverage on schistosomiasis prevalence or intensity.

**Fig. 4:**
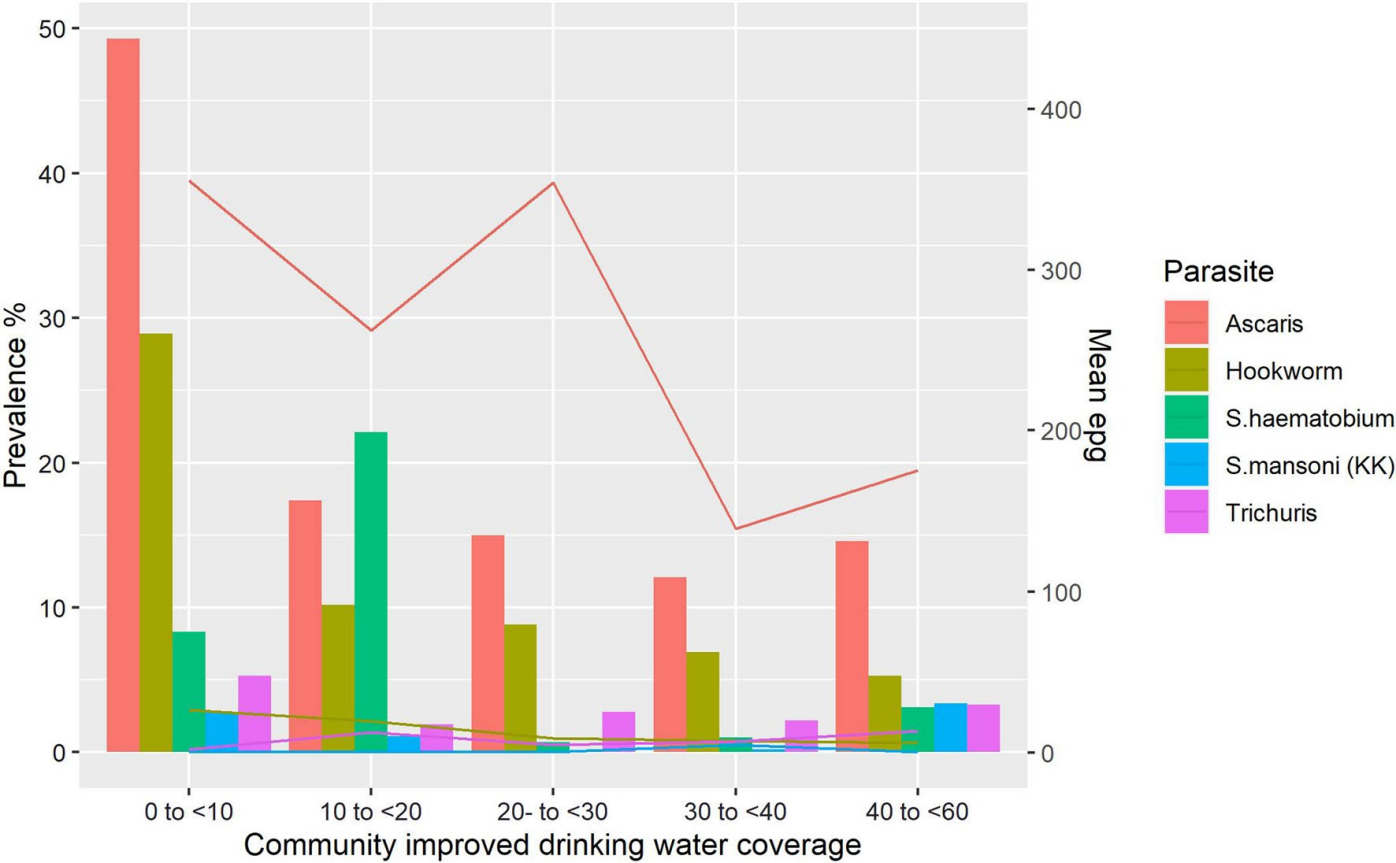
Prevalence of STH and schistosomiasis infection by community access to improved drinking water

Figure 4 (and Additional file 1: Table S2) examines the association of community access to improved drinking water. Again, significantly higher prevalence and intensity of both STH and schistosomiasis infection were seen among communities where improved drinking water coverage was < 20%.

Table 6 shows odds ratios in strata of community sanitation usage and household latrine coverage (by JMP category) with infection. The odds of *A. lumbricoides* infection decreased as community sanitation coverage improved to 20–40% (OR: 0.63) and further as coverage was > 40% (OR: 0.35). Likewise, odds of *T. trichiura* infection increased significantly when community sanitation was < 40% (OR: 3.2). Hookworm prevalence, however, was significantly modified by household sanitation where infection increased with limited sanitation (OR: 1.49) and further still with unimproved latrine access (OR: 1.56).

**Table 6:** Logistic regression of community and household WaSH coverage by STH infection

Parasite	Sanitation		OR	95% CI	*P*	AOR*	95% CI	*P*
*A. lumbricoides*	Community	< 20%	1.1	0.9, 1.2	0.6	1.1	0.9, 1.2	0.5
		20–40%	0.6	0.5, 0.8	< 0.01	0.6	0.5, 0.8	< 0.01
		> 40%	0.4	0.2, 0.5	< 0.01	0.4	0.2, 0.5	< 0.01
	Household	Basic	1.0	0.7, 1.4	0.9	1.2	0.9, 1.7	0.3
		Limited	1.0	0.7, 1.5	1.0	1.3	0.8, 2.0	0.3
		Unimproved	1.1	0.8, 1.4	0.5	1.1	0.8, 1.4	0.5
*T. trichiura*	Community	< 20%	3.2	2.2, 4.6	< 0.01	3.3	2.2, 4.8	< 0.01
		20–40%	1.3	0.7, 2.3	0.4	1.4	0.8, 2.6	0.2
		> 40%	0.5	0.2, 1.4	0.2	0.6	0.2, 1.6	0.8
	Household	Basic	0.9	0.4, 2.0	0.8	0.9	0.4, 2.0	0.8
		Limited	1.0	0.4, 2.7	1.0	1.0	0.3, 2.6	0.9
		Unimproved	1.4	0.7, 2.5	0.4	1.4	0.7, 2.5	0.4
Hookworm	Community	< 20%	1.2	1.0, 1.4	0.2	1.1	1.0, 1.3	0.2
		20–40%	0.8	0.6. 1.0	0.07	0.8	0.6. 1.1	0.2
		> 40%	0.9	0.7, 1.3	0.7	1.0	0.7, 1.3	0.8
	Household	Basic	1.1	0.7, 1.9	0.7	1.2	0.7, 2.0	0.6
		Limited	1.5	1.0, 2.2	0.05	1.6	1.0, 2.3	0.03
		Unimproved	1.6	1.1, 2.2	0.01	1.6	1.1, 2.2	0.01

^*^Variables for multivariable analysis were selected using an inclusion criterion of *P*-value < 0.1 in a sequential (block-wise) variable selection method; however, sex and age were retained as fixed terms in the final models regardless of statistical significance because of their known importance

## Discussion

This is a baseline assessment of the association between WaSH access and STH/schistosomiasis infection in Wolayita zone, Ethiopia. The overall prevalence of both STH and schistosomiasis in the present study was low (with *A. lumbricoides* at 9.5%, 7.2% for hookworm, 1.8% for *T. trichiura,* 0.1% *S. haematobium* by urine filtration, and 0.8% *S. mansoni* prevalence by Kato-Katz). Both STH and schistosome infections were highly aggregated as is typical for helminth parasite infections, with few individuals releasing a disproportionately high number of eggs. This region has received multiple rounds of treatment through school-based deworming since 2015; therefore, the parasite aggregation may be a result of poor adherence to treatment in a subset of the population [44]. Persistent non-compliance to treatment within a community, particularly when WaSH access is poor, will maintain a reservoir of infection thereby preventing the possibility of transmission cessation through PC alone.

Our analyses reveal some important results on the impact of access to improved community and household WaSH infrastructure on STH and schistosomiasis infection levels. The findings report an association between infection among a cross-section of age groups prior to the start of any interventions. Several household WaSH characteristics were found to be significantly associated with *A. lumbricoides* and hookworm infection. Specifically, increased time taken to fetch water, sharing a latrine, and lack of handwashing facilities were all statistically associated with increased infection. It is likely that households far from central water access points are more remote and may also be less like to receive social mobilisation messages about WaSH, BCC, and PC. Past work has also shown that sharing latrines between families is associated with an increased risk of helminth infection [45] and as a result is included in the JMP service ladder [40]. Latrine cleanliness, which may be harder to maintain when shared, may contribute particularly to the risk of hookworm transmission. Due to the exposure pathway for *A. lumbricoides* from ingestion of faeces on hands or eating unwashed food, it was expected to see an association with lack of handwashing facilities. We found no evidence of WaSH coverage on *T. trichiura*, likely due to the few individuals infected in the sample analysed.

Since hookworm is transmitted through direct contact of infected faeces and soil-based larvae with bare feet, our results reflect the common finding that individuals who do not wear shoes are more susceptible to hookworm infection. Shoe wearing is likely to be associated with socioeconomic status, a factor not addressed in this analysis, but may moderate the effect of any WaSH interventions.

Like STH, households that took longer to collect drinking water, sharing a latrine, and with lack of handwashing facilities were significantly associated with schistosomiasis. As expected, the main age group affected by schistosome infections was SAC. Schistosome infection was also higher among males, likely because of greater recreational water contact. There was also a significant association between greater risk of schistosome infection and infant faeces not being disposed from the household environment. This may be from mothers washing nappies in freshwater bodies or children defecating in water when playing. This highlights the importance of infant sanitation interventions being incorporated into WaSH interventions, an element that is often overlooked. Like STH, there was also an increased risk of schistosome infection in households without handwashing facilities. Unsurprisingly recreational bathing or clothes washing was significantly associated with schistosome infection.

Aggregating household WaSH access at the community level was helpful to shed light on the threshold at which community coverage has an impact on STH and schistosome infection. These findings indicate that household access to a latrine may only be beneficial for reducing STH and schistosomiasis if the wider community, specifically a threshold > 20% community coverage, has access to improved sanitation. It is widely accepted that community access to improved latrines and drinking water is needed [46], and these results show coverage had significant impact on both STH and schistosomiasis, respectively. Often studies focus solely on individual access to WaSH at the household level, but the impact of the community as a whole is clearly very important and hints at the importance of community-based reservoirs of infective stages for all individuals in a given setting.

## Strengths and limitations

Some limitations should be considered when interpreting the results of this research. Assessing the impact of WaSH interventions on infections such as STH and schistosomiasis faces several challenges. These include the lack of sufficient study duration to gauge long-term impact of behavioural change and the reliance on self-reporting behaviour (potentially introducing social desirability bias). Furthermore, the provision of latrines is only part of the improvements necessary to restrict infection; good maintenance is also required. Many low-cost latrines fall short of basic standards because emphasis is on coverage rather continued maintenance, which if done well, encourages continued use. Future studies should explore numbers of individuals sharing latrines, as well as quantifying latrine maintenance and cleanliness scores, and the moderating effect of shoe wearing (hookworm only) to explore possible transmission pathways.

The use of Kato-Katz as the primary diagnostic for STH and *S. mansoni* is a limitation of this study, as the sensitivity of Kato-Katz, particularly in low prevalence settings, is known to be poor [47]. Nevertheless, the baseline mapping sampled 40% of communities in Wolayita, providing a highly granular picture of helminth infection prevalence across the zone.

Relatively few studies have investigated the impact of school-level WaSH coverage on helminth infections. A cluster-randomized trial in Kenya found that the provision of school-based WaSH improvements may reduce reinfection of STHs after school-based deworming, but the magnitude of the effects may be sex and helminth species specific [48]. Grimes et al. constructed scores reflecting exposure to schistosomes arising from (fresh) water collection for schools and the adequacy of school sanitation and hygiene facilities [49]. The findings were that improving school WaSH may reduce transmission of both STH and schistosomiasis; however, the impact of the different WaSH interventions was parasite dependent. In particular, the strongest associations were between water and *S. mansoni*, sanitation and *A. lumbricoides*, and hygiene and hookworm. Data were collected at the school level for the Geshiyaro project and will be analysed in a future publication.

The WHO/UNICEF JMP criteria are useful to explore associated risk of STH/schistosomiasis infection with each tier of the service ladder. Water quality was not considered, yet it is of great importance to understand whether chlorination is as effective against schistosome cercariae and STH eggs as it is for coliform bacteria. In this study, self-reported use of chlorination of drinking water was associated with a decreased risk of STH and *S. mansoni*. Environmental influences such as floods and heavy rains can also exacerbate transmission both within and between communities, factors not considered in this analysis. Zoonotic infection via contact with animal faeces also requires consideration in future studies given the mounting evidence that human schistosome parasites and *A. lumbricoides* can be zoonotic in livestock [50, 51]. Finally, insufficient data were available in the datasets to be able to determine safely managed sanitation by the JMP definition.

A major strength of this study was the ability to link individual (through a study ID card or biometric) WaSH access (at both the individual, household, and community-level) with their parasitological results. This triangulation of data across surveys strengthens the validity of the results. Furthermore, a large number of participants have been enrolled in the project, all of whom were selected through population-based recruitment, which improves the generalisability of the findings.

## Conclusions

When scaling down a helminth NTD control programme predominantly based on deworming, it is widely accepted that WaSH infrastructure and good practices need to be in place to ensure that the control/elimination is sustained when treatment ceases. Ideally these WaSH improvement measures should be solely sufficient in the longer term to reduce the basic reproductive numbers of these helminth infections below unity in value. In combination with PC, they act to enhance the impact of treatment by reducing the effective reproductive number R. These results show the impact of both community and household risk WaSH coverage on an individual’s risk of infection. Measuring that impact can be difficult as uptake can vary greatly person by person, household by household, and community by community, as clearly illustrated in this study. The most important finding from the analyses was the effect of community-level WaSH factors on all individuals in that community. The great heterogeneity between individuals can mask the detection of WaSH impact, but at a community level it is apparent.

Once treatment ceases, bounce back in infection can occur very rapidly and hence gains made from PC are difficult to sustain if treatment is not repeated frequently. This paper suggests that there is an obvious need to augment PC with WaSH in preventing reinfection. However, more guidance on best-practice sanitation and hygiene promotion approaches relevant to the disease context and programme location is needed. An integrated strategy including guidelines with disease-specific indicators and targets, at both the household and community level, will benefit helminth control and contribute to the evidence base of how best, and cost effectively, to implement helminth NTD control programmes.

## Supplementary Information

**Additional file 1: Table S1**. Prevalence of STH and schistosomiasis infection by community sanitation coverage. **Table S2**. Prevalence of STH and schistosomiasis infection by community access to improved drinking water.

## Data Availability

The datasets generated and/or analysed during the current study are available from the corresponding author upon reasonable request.

## Abbreviations

ALB: Albendazole
BCC: Behavioural change communication
CI: Confidence intervals
CLTS: Community-led total sanitation
EPHI: Ethiopian Public Health Institute
JMP: Joint Monitoring Programme for Water Supply and Sanitation
NTDs: Neglected tropical diseases
OWNP: One WaSH National Programme
ODF: Open defecation free
Pre-SAC: Pre-school aged children
PC: Preventive chemotherapy
POC-CCA: Point-of-care circulating cathodic antigen
PZQ: Praziquantel
PC: Preventive chemotherapy
RCT: Randomised controlled trials
SAC: School-aged children
STH: Soil-transmitted helminths
WaSH: Water, sanitation, and hygiene
WHO: World Health Organisation
