# Supplementary material for: Association between water, sanitation, and hygiene access and the prevalence of soil-transmitted helminth and schistosome infections in Wolayita, Ethiopia
Source: Parasit Vectors. 2022 Nov 4;15:410. doi: 10.1186/s13071-022-05465-7 (PMC9636783; doi:10.1186/s13071-022-05465-7)
Supplement: Supplementary file 1 — Additional file 1: Table S1. Prevalence of STH and schistosomiasis infection by community sanitation coverage. Table S2. Prevalence of STH and schistosomiasis infection by community access to improved drinking water. [file 13071_2022_5465_MOESM1_ESM.docx]

**Supplementary Table S1. Prevalence of STH and Schistosomiasis infection by community sanitation coverage**

|  |  | **% (95% CI) households in a community with improved sanitation** | | | | | |
| --- | --- | --- | --- | --- | --- | --- | --- |
| **Characteristics** | **N** | **0 to <10** | **10 to <20** | **20 to <30** | **30 to <40** | **40 to<50** | **P** |
| No. individuals | 6,681 | 2,474 | 2,516 | 932 | 518 | 241 |  |
| No. communities | 53 | 14 | 19 | 4 | 4 | 2 |  |
| *A. lumbricoides* prevalence | 916 | 15.1 (13.7, 16.5) | 15.7 (14.3, 17.1) | 10.1 (8.3, 12.2) | 5.8 (4.1, 8.2) | 6.4 (3.1, 10.9) | **<0.01** |
| *A. lumbricoides* mean epg |  | 255.7 (204, 307) | 267.6 (220, 314) | 69.9 (37.1, 102) | 29.1 (7.5, 50.8) | 85.7 (13.0, 158) | **<0.01** |
| *T. trichiura* prevalence | 178 | 1.50 (1.1, 2.1) | 4.57 (3.8, 5.5) | 1.93 (1.2, 3.1) | 0.77 (0.3, 2.) | 0.66 (0, 3.4) | 0.73 |
| *T. trichiura* mean epg |  | 12.6 (3.4, 21.8) | 16.9 (9.3, 24.4) | 12.5 (0, 35.3) | 8.86 (0, 25.0) | 21.5 (0, 51.1) | **<0.01** |
| Hookworm prevalence | 689 | 10.2 (9.1, 11.5) | 11.5 (10.3, 12.8) | 8.15 (6.6, 10.1) | 9.65 (7.4, 12.5) | 7.71 (4.7, 12.0) | 0.17 |
| Hookworm mean epg |  | 19.9 (11.1, 28.8) | 16.7 (11.7, 21.8) | 7.44 (4.7, 10.2) | 16.1 (8.0, 24.2) | 11.5 (4.9, 18.1) | **<0.01** |
| Any STH prevalence | 1,518 | 23.6 (22.0, 25.4) | 25.6 (24.0, 27.4) | 18.0 (15.7, 20.6) | 15.1 (12.2, 18.4) | 15.4 (11.1, 20.8) | **<0.01** |
| *S.mansoni* (KK) prevalence | 104 | 1.17 (0.8, 1.7) | 2.31 (1.8, 29.7) | 0.75 (0.4, 1.6) | 1.93 (1.0, 3.6) | 0 | 0.62 |
| *S.mansoni* (KK) mean epg |  | 0.33 (0.1, 0.5) | 1.14 (0.8, 1.5) | 6.71 (0.5, 19.2) | 0.74 (0.2, 1.2) | 0 | 0.59 |
| *S.mansoni* (POCTr+) | 844 | 19.2 (17.3, 21.2) | 22.5 (20.6, 24.5) | 13.3 (10.7, 16.3) | 36.4 (29.1, 44.4) | 14.0 (9.2, 20.7) | 0.99 |
| *S.haematobium* | 185 | 2.42 (1.9, 3.1) | 3.66 (29.8, 4.5) | 2.41 (1.6, 3.6) | 1.33 (0.6, 2.9) | 4.14 (2.2, 7.5) | 0.87 |

**Supplementary Table S2. Prevalence of STH and Schistosomiasis infection by community access to improved drinking water**

|  |  | **% (95% CI) households in a community with access to improved drinking water** | | | | | |
| --- | --- | --- | --- | --- | --- | --- | --- |
| **Characteristics** | **N** | **0 to <10** | **10 to <20** | **20- to <30** | **30 to <40** | **40 to <60** | **P** |
| No. individuals | 6,681 | 2,474 | 2,516 | 932 | 518 | 241 |  |
| No. communities | 53 | 14 | 19 | 4 | 4 | 2 |  |
| *A. lumbricoides* prevalence | 916 | 49.3 (31.3, 77.9) | 17.4 (15.1, 19.9) | 15.0 (12.4, 18.1) | 12.1 (8.9, 16.2) | 14.6 (13.1, 16.3) | **<0.01** |
| *A. lumbricoides* mean epg |  | 355.5 (217, 466) | 262.3 (167, 357) | 354.3 (242, 466) | 139.0 (36.6, 241) | 175.4 (137, 213) | **<0.01** |
| *T. trichiura* prevalence | 178 | 5.3 (3.8, 7.4) | 1.9 (1.2, 2.9) | 2.8 (3.8, 17.7) | 2.2 (1.1, 4.6) | 3.3 (2.6, 4.2) | 0.74 |
| *T. trichiura* mean epg |  | 1.7 (0, 4.9) | 12.4 (1.1. 23.7) | 4.6 (0, 9.7) | 6.6 (2.4, 10.7) | 13.1 (6.9, 29.6) | **0.01** |
| Hookworm prevalence | 689 | 28.9 (20.6, 40.4) | 10.2 (7.27, 14.0) | 8.8 (7.2, 10.7) | 6.9 (5.9, 8.1) | 5.27 (3.3, 7.4) | **<0.01** |
| Hookworm mean epg |  | 26.1 (5.6, 46.5) | 19.2 (4.1, 34.2) | 8.9 (6.1, 11.7) | 7.1 (3.8, 10.4) | 5.84 (3.6, 8.1) | **<0.01** |
| *S.mansoni* (KK) prevalence | 104 | 2.8 (0.38, 17.7) | 1.1 (0.6, 2.0) | 0 | 0.2 (0, 1.2) | 3.37 (2.7, 42.8) | 0.39 |
| *S.mansoni* (KK) mean epg |  | 0.3 (0, 1.0) | 0.4 (0.1, 0.7) | 0.3 (0, 1.0) | 4.5 (0, 10.5) | 0.22 (0.1, 0.4) | **0.01** |
| *S.mansoni* (POCTr+) | 844 | 8.5 (6.4, 11.2) | 28.9 (19.8, 40.2) | 28.9 (26.3, 31.7) | 15.3 (10.3, 22.1) | 18.5 (16.6, 20.4) | **<0.01** |
| *S.haematobium* | 185 | 8.3 (2.7, 23.2) | 22.1 (14.5, 33.8) | 0.7 (0.2, 2.7) | 1.0 (0.4, 2.2) | 3.1 (2.4, 4.0) | **<0.01** |
